# Plasma Cells, the Next Generation: Beyond Antibody Secretion

**DOI:** 10.3389/fimmu.2019.02768

**Published:** 2019-11-22

**Authors:** Peter D. Pioli

**Affiliations:** Department of Biomedical Sciences, Center for Immunobiology, Western Michigan University Homer Stryker M.D. School of Medicine, Kalamazoo, MI, United States

**Keywords:** plasmablasts, plasma cells, inflammation, hematopoiesis, Toll-like receptor, immunoglobulin

## Abstract

Plasma cells (PCs) represent the terminal differentiation step of mature B lymphocytes. These cells are most recognizable for their extended lifespan as well as their ability to secrete large amounts of antibodies (Abs) thus positioning this cell type as a key component of humoral immunity. However, it is now appreciated that PCs can have far reaching effects on pathologic as well as non-pathologic processes independent of Ab secretion. This is highlighted by recent studies showing that PCs function as key regulators of processes such as hematopoiesis as well as neuro-inflammation. In part, PCs accomplish this by integrating extrinsic signals from their environment which dictate their downstream functionality. Here we summarize the current understanding of PC biology focusing on their ever-growing functional repertoire independent of Ab production. Furthermore, we discuss potential applications of PC immunotherapy and its implementation for translational benefit.

## Introduction

Trademarks of the adaptive immune system include the ability to respond to diverse sets of antigenic stimuli and the long-term durability of this response. The former is initiated early in B and T lymphocyte development and is driven by V(D)J recombination of antigenic gene loci (1). In the case of B lymphocytes, this antigenic diversity can be further modified via somatic hypermutation (SHM) (2) and class switching (3) during a subsequent immune response. In contrast, the durability of an immune response results from the formation of long-term immunological “memory” which includes cell types such as memory B and T lymphocytes (4) as well as plasma cells (PCs) (5).

A significant goal upon vaccination or infection is the production of protective Abs for a sustained period of time. Given this, it can be rationally perceived that PCs sit at the apex of adaptive immunity in the sense that these cells have the potential to survive indefinitely in both mice (6–9) and humans (10–12) while also continuously secreting Abs (9). This latter phenotype being thanks in part to an expanded Golgi-network that provides PCs with their signature peri-nuclear halo when viewed under a microscope (13). As such, the accumulation of these cells over a lifetime potentially represents a historical record of humoral immune responses. Not surprisingly, numerous studies have focused on the induction of PCs differentiation from the perspective of generating antigen-specific Ab responses (14).

However, the field has recently begun to appreciate the multitude of functions that PCs possess aside from Ab secretion (15–18). This Perspective highlights the evolving functions of PCs and discusses the potential for environmental interactions to program these diverse regulatory behaviors. Furthermore, we consider the long-term effects of these cells on various biological processes including aging and introduce strategies that may become key means of modulating PC functionality for a beneficial outcome.

## More than Just Antibody Factories

### Regulation of Infectious and Autoimmune Immune Responses

**Figure 1:**
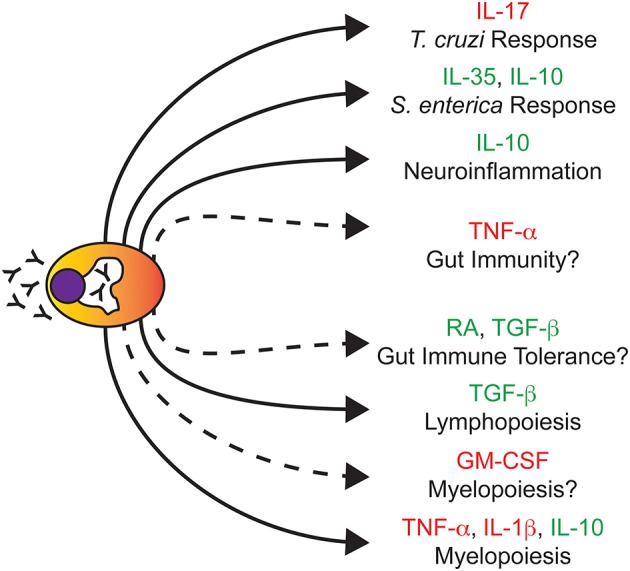
Plasma cells regulate biological processes independent of immunoglobulins. Due to their enhanced endoplasmic reticulum-Golgi structure (white peri-nuclear halo), PCs are best known for their ability to secrete Ig (depicted as Y). Illustrated here are various non-Ig secreted factors that PCs produce and the biological processes that PCs are known to regulate. Solid connections represent studies in which the removal of PCs and/or their secreted factors had a documented biological outcome. Dashed connections represent predicted regulatory nodes based on the cytokines produced which require further experimental validation. Cytokines highlighted in red or green are commonly associated with being pro-inflammatory or anti-inflammatory, respectively. Note that this figure does not summarize studies of Ig-based PC effector function.

For decades, it has been presumed that upon maturation, PCs migrate to the bone marrow (BM) where they remain quiescent and ONLY secrete copious amounts of Abs. However, this viewpoint has now been challenged (Figure 1). In a landmark study, it was shown that stimulation of mice with lipopolysaccharide (LPS) led to the generation of a population of granulocyte-macrophage colony-stimulating factor (GM-CSF) producing cells referred to as innate response activator (IRA) B cells (19). Phenotyping of IRA B cells demonstrated the expression of the PC marker, CD138. Transcriptional analysis of these cells further supported the idea that IRA B cells may be a *bona fide* subset of PCs as their gene signature clustered closest to PCs compared to other B cell subsets. Similarly, recent studies in mice examining responses to infectious agents such as *Trypanosoma cruzi* and *Salmonella enterica* have demonstrated the propensity of PCs to express cytokines such as interleukin (IL)-17 (20) as well as IL-35 and IL-10 (21), respectively. In this regard, B lymphocyte derived IL-17 was absolutely required for efficient control of *T. cruzi* infection and dampening of infection-associated inflammation following pathogen clearance (20). In contrast, B lymphocyte-derived IL-35 was detrimental in the context of *Salmonella* infection as its deletion led to enhanced monocyte and T lymphocyte responses upon infection (21). While these studies focused on B lymphocyte-specific cytokine ablation and not just PC-specific cytokine deletion, they clearly demonstrated that factors produced by PCs as well as other B lymphocyte populations play a critical role in regulating host-pathogen interactions.

The role of PCs in the progression of autoimmune disease is well-known with the focus mainly on Ab production as auto-Abs, through their constant regions, can potentially induce a pro-inflammatory cascade (22). Studies in mouse models of lupus (23–26) as well as human systemic lupus erythematosus (SLE) patients (27) have shown that PC depletion reduces the level of autoreactive antibodies as well as disease burden. However, the role of PCs in autoimmunity is not solely limited to SLE (28).

A recent study elegantly demonstrated the role of IL-10 producing PCs in the suppression of neuroinflammation in a mouse model of autoimmune encephalomyelitis (EAE) which recapitulates some features of multiple sclerosis (MS) in humans (18). Using genetic models combined with BM chimeras, the authors demonstrated that in this instance, PC-produced IL-10 was the key molecule in the suppression of EAE-induced pathology. These IL-10-producing PCs were originally derived from the small intestine and migrated to the central nervous system (CNS), an important observation given the association of the microbiota with diseases such as multiple sclerosis (29). Using *Mb-1*-Cre-mediated deletion of *Prdm1* to ablate PC differentiation, a previous report also observed a critical role for PCs in the suppression of EAE in mice (30). In this instance, *in vitro* co-culture experiments implicated a role for PB-derived IL-10 in the suppression of dendritic cell function and subsequent interferon-gamma (IFN-γ) production by CD4^+^ T cells. These results point to PCs having a beneficial effect in the context of EAE. However, this is not the case for all autoimmune disorders of the nervous system. For instance, neuromyelitis optica (NMO) targets the optic nerve and spinal cord resulting in their degeneration and this is largely thought to be due to the production of auto-Abs targeting aquaporin 4 (AQP4), which is highly expressed in the central nervous system (CNS) (31, 32). Direct evidence of this was demonstrated in a in a rat model in which AQP4-specific auto-Abs cloned from human patients induced overt NMO pathology highlighted by astrocyte depletion and myelinolysis following their administration (33). Another study demonstrated the reliance of PBs isolated from human NMO patients on the cytokine IL-6 for not only enhanced survival but AQP4 auto-Ab secretion as well (32). Whether or not PBs in NMO can be an autocrine supply of this cytokine becomes an interesting question in light of *in vitro* experiments that demonstrated the capacity of human PBs to produce IL-6 (34). In the studies of EAE noted above, it is unclear whether or not the responding PCs were clonally derived; that is, antigen specific as is evident in the NMO studies described here as well as in human cases of multiple sclerosis (35).

### Regulation of Hematopoiesis by Plasma Cells

In BM, PCs localize in close proximity to stromal cells with ~80% of BM PCs directly contacting stroma (36). While being a key component of the PC survival niche (37), stromal cells are also essential in the proper maintenance of hematopoiesis (38). Therefore, it stands to reason that PCs have the potential to regulate hematopoiesis directly through actions on progenitors or indirectly by regulating the stromal niche. Indeed, this is the case as we identified PCs as key effectors driving enhanced myelopoiesis observed in aging mouse BM (17). Ab-mediated depletion of PCs in aged mice led to the reduction of myelopoiesis to levels observed in young animals in part through alterations in the BM niche highlighted by reduced expression of myeloid promoting factors such as *Il1b* and *Csf1* by BM stromal cells. Co-culture experiments demonstrated that the ability of PCs to drive increased myelopoiesis was age-dependent as young PCs did not promote myelopoiesis in contrast to old PCs. Transcriptional profiling via RNA-sequencing (RNA-seq) demonstrated that old PCs adopted an inflammatory gene signature which could be exacerbated by Toll-like receptor (TLR) stimulation as evidenced by increased *Il1b* and *Tnf* gene expression following *in vitro* lipopolysaccharide (LPS) stimulation. Pharmacological blockade of IL-1 and tumor necrosis factor-alpha (TNF-α) signaling reduced granulopoiesis in aged mice demonstrating the importance of these factors in the age-associated increase in myelopoietic output (17). Notably, a recently published study (16) demonstrated that *in vitro* derived mouse PCs had the potential to regulate the composition of myeloid cells generated in culture. Here, IL-10 was the critical regulator that skewed the myeloid compartment toward a more macrophage-like cell fate (16).

In regards to lymphopoiesis, both mouse (17) and human (39) PCs have been shown to suppress B lymphopoiesis. In the latter study, the ability to inhibit B lymphopoiesis required interactions between multiple myeloma (MM) cells, a PC-derived neoplasm, and stromal cells which led to increased expression of *CCL3* (macrophage inflammatory protein-one alpha, MIP-1α) and C*CL4* (macrophage inflammatory protein-one beta, MIP-1β) by stromal cells as well as increased expression of *TGFB1* (transforming growth factor-beta one, TGF-β1) by both PCs and stromal cells (39). Addition of either TGF-β1 or MIP-1β to pre-B lymphocyte cultures was sufficient in suppressing B lymphopoiesis *in vitro* demonstrating the regulatory potential of these factors (39). Collectively, these studies demonstrate the ability of PCs to regulate a fundamental process in hematopoiesis. Furthermore, the data suggest that PCs do so independently of their ability to produce Abs and in at least some instances, a cytokine-dependent manner.

### Gut Homeostasis and Plasma Cells

A majority of PCs reside in the gut as this tissue provides a direct interface between host cells and microbes (40). These cells play critical roles in gut homeostasis through the production of secretory IgA (SIgA) (41) which regulates not only exclusion of IgA-cross reactive bacteria but also the propagation of the immune response (42). However, a recent study in mice (15) has shown that PCs promote the generation of regulatory T (T_Reg_) cells in the gut through the production of TGF-β and retinoic acid (RA) suggesting the importance of PCs in maintaining intestinal immune tolerance. Paradoxically, PCs from the murine lamina propria (43) have also been shown to produce TNF-α as well as inducible nitric oxide synthase (iNOS), a key regulator of IgA class switching (44). It remains to be determined if the same PCs have the potential to make TGF-β, RA, and TNF-α or alternatively, if these represent unique cytokine-producing PC subsets. How the relative balance of anti-inflammatory (TGF-β, RA) and pro-inflammatory (TNF-α) cytokine production in the gut alters its homeostasis is a critical question given the potential roles of these cytokines in pathologies such as intestinal bowel disease (45).

## Programming Plasma Cell Function

### Plasma Cells Express Functional Membrane-Associated Immunoglobulins

Previous assumption has been that PCs do not express membrane immunoglobulin (mIg). We now know differently as IgM^+^ and IgA^+^ PCs in both mouse (17, 46) and human (47) have been shown to express mIg. This is true for IgE^+^ PCs in mice as well (48). Multiple studies have demonstrated that these surface BCRs are fully functional. In humans, Ig crosslinking of mIgM^+^ and mIgA^+^ PCs led to increased amounts of phosphorylated Syk as well as phosphorylated ERK and AKT which represent events proximal and distal to BCR signaling, respectively (47). Given the importance of these kinases in B lymphocyte viability, it is not surprising that low dose mIg crosslinking provided a survival benefit to mIgA^+^ PCs *in vitro* (47). *In vivo*, a recent murine study showed that stimulation of BM mIgM^+^ PCs through their mIg led to increased surface expression of CD69 as well as changes in gene expression highlighted by increases in the aforementioned *Il10* as well as *Ccl5* (46), a pro-myelopoietic cytokine (49). Taken together, these data suggest that mIg activation of a previously established pool of PCs may elicit a pro-myelopoietic transcriptional response; however, experiments are needed to validate this hypothesis.

### Plasma Cells Are Responsive to Toll-Like Receptor Signals

TLR signaling in B lymphocytes contributes to the formation of the PC compartment (50, 51). Not unlike B lymphocytes, terminally differentiated PCs also express TLRs. In humans, PCs isolated from the peripheral blood as well as the tonsils have been shown to express TLRs 1–10 (52). Additionally, the stimulation of human PCs with TLR ligands such as peptidoglycan, poly(I:C) and flagellin significantly increased Ab secretion *in vitro* (52). Similarly, TLR expression has been detected in human MM both in cell lines and primary cells (53, 54). In regard to MM, TLR signals have been observed to provide context dependent signals. For example, TLR4 ligation enhanced survival and proliferation (54, 55) whereas TLR1/2 derived signals sensitized MM cells to chemotherapy-induced cell death (53).

However, the classical roles of TLR signaling respective to inflammatory responses remain understudied in both human and mouse PCs. Mentioned previously, PCs isolated from the BM of old mice displayed increased levels of *Tlr4* gene expression and were highly responsive to LPS signaling in contrast to their young counterparts (17). Additionally, old murine PCs displayed increased expression of *Tlr6, Tlr7, Naip2, Naip6*, and *Nod2*. It will be interesting to determine if these signaling pathways are also functionally responsive in PCs and to what biological outcome.

## Selective Elimination of Plasma Cells for Translational Outcomes

It is becoming readily apparent that PCs play significant roles in biology outside of Ab secretion. However, the full spectrum of PC functionality has yet to be defined. In this sense, it will be important to determine the causes, or context dependent signals, that drive plasma cell heterogeneity such as the surrounding cytokine milieu and even the nature of the antigen itself. Indeed, phenotypically long-lived PCs have been identified in various organs in both rodents (56–59) and humans (11, 12, 60) however, it is not fully understood how these differing niches may regulate PC behavior. Furthermore, whether these cues act directly on PCs themselves as has been demonstrated for the model antigen 4-hydroxy-3-nitrophenylacetic-dextran (NP-dextran) (46) or if PC phenotypes are imprinted from the activated B cell that lies upstream will be important to consider. With that being said, our recent study regarding PCs and their effects on age-associated patterns of hematopoiesis in mice (17) provides an example of how PCs can be targeted for a potential therapeutic benefit. Aging is associated with increases in myeloid leukemias which has been correlated with the heightened levels of BM myelopoiesis also observed with age (61, 62). In both humans (63) and mice (64, 65), this is initiated at the most primitive progenitor levels as the hematopoietic stem cell (HSC) compartment becomes more myeloid-biased with age. At least in mice, this is in part due to the preferential expansion of myeloid-biased HSCs (My-HSCs) rather than the loss of HSCs with inherent lymphoid-bias (Ly-HSCs) (66).

Using Abs directed toward CD138, a cell surface determinant common to PCs, we successfully depleted their numbers and reversed the expanded myelopoiesis commonly associated with aging (17). While this strategy accomplished its goal and may potentially be a route toward reducing the risk of age-associated myeloid leukemias, it is not feasible in “real life.” The reason being is that PCs are the major Ab producers in the body and theoretically, provide protective immunity derived from even the earliest of ages. As such, depleting the bulk, if not all, of the PC pool would leave the host severely immunocompromised. This may not be as critical in younger individuals as they generally possess robust immune responses and can be re-vaccinated to restore the PC pool once a particular translational goal is achieved. However, the elderly are more susceptible to infection and possess weakened responses to vaccination (67). In part, this is due to age-associated changes in B lymphocytes which include an altered immune repertoire as well as decreased expression of E2A and activation-induced cytidine deaminase (AID) which would be predicted to compromise PC differentiation (68).

**Figure 2:**
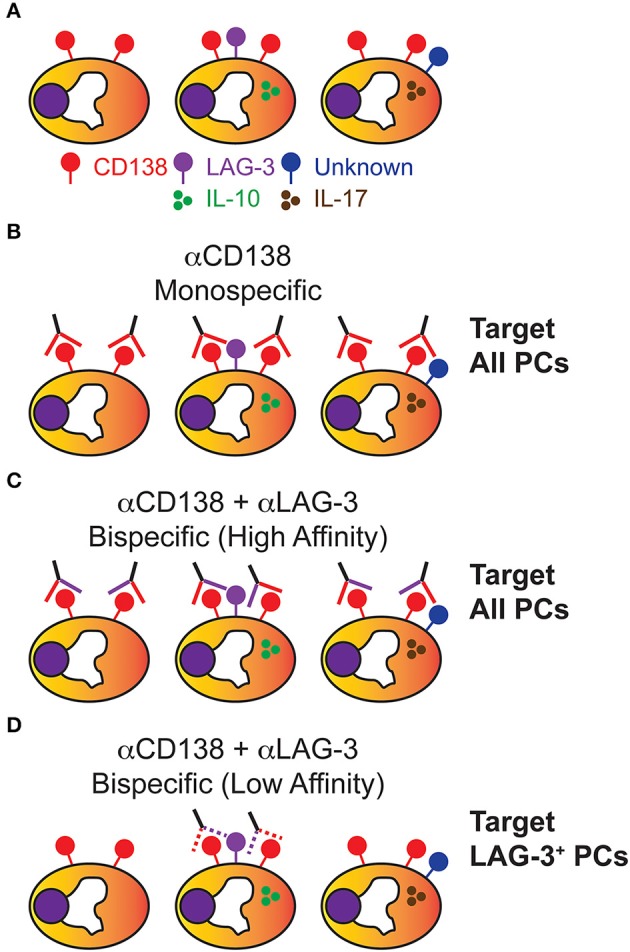
Plasma cells are heterogenous and can be potentially targeted in a subset specific manner. **(A)** While all PCs express the cell surface determinant CD138, PCs are heterogenous in the cytokines produced and various cell surface markers they express, of which only some are known. **(B)** Using αCD138 antibodies, PCs can be homogenously targeted for depletion which randomly and significantly depletes the PC-derived Ig pool. **(C)** Using a high affinity bispecific antibody designed to react against CD138 and LAG-3, all PCs are presumably depleted due to strong CD138 reactivity similar to **(B)**. **(D)** Using a low affinity bispecific antibody designed to react against CD138 and LAG-3, a therapeutic window has now been theoretically created which allows for the specific depletion of IL-10-producing CD138^+^ LAG-3^+^ PCs while still leaving the rest of the PC Ig repertoire intact.

For PC depletion/modulation to be a successful strategy, the PC pool must be heterogenous in nature and possess cell surface determinants specific for a given PC subpopulation (Figure 2A). To some degree, we already know this to be true. For example, IL-17 producing PCs generated following *T. cruzi* infection only constitute roughly ~6–8% of the total splenic PC pool at 10 days post-infection (20). Using *Il10*eGFP reporter mice (69), it was shown that LAG-3 was sufficient to prospectively identify *Il10*eGFP^+^ PCs which constituted approximately 20 and 40% of total PCs in BM from young and old mice, respectively. Thus, functional PC subsets exist and, in some instances, can be identified via expression of unique cell surface proteins.

So how do we ablate a particular subset such as LAG-3^+^ PCs? Cell type-specific targeting would be key as depleting or altering the function of all LAG-3^+^ cells would potentially lead to the loss of tolerance through adverse effects on the T_Reg_ pool (70). Looking toward the field of tumor immunotherapy (71), we find that a potential solution would be the generation of bispecific Abs reactive to both CD138 and LAG-3 which would preferentially target IL-10-producing PCs (Figures 2B–D). Having a high affinity bispecific Ab would be favorable in a tumor setting where elimination of every cell is the desired outcome. However, in terms of targeting a particular subset of PCs, this would still result in targeting all PCs through efficient binding of CD138 (Figure 2C). One would need to develop a bispecific Ab where each epitope is bound sub-optimally and thus only PCs which expressed both CD138 and LAG-3 would be bound with high enough affinity required for depletion (Figure 2D).

Secondarily, the mode of elimination would need to be chosen in an application specific manner. For example, these Abs could be conjugated to a particular toxin such as type I interferon or even bortezomib, a MM chemotherapeutic. However, it is not known if this strategy would cause high levels of local or even systemic inflammation which could have unintended consequences. For example, lymphopoiesis is suppressed by high levels of inflammatory cytokines such as IL-1 while in contrast, inflammation enhances myelopoiesis (72). As such, using an unconjugated Ab may be most prudent. Mechanistically it is not known how unconjugated CD138 antibodies deplete PCs (17). This could be through Ig constant region-mediated interactions such as with complement or even through the deprivation of survival signals as CD138 has been demonstrated to promote PC cell survival through heparan sulfate-mediated interactions with IL-6 and APRIL (73). In this regard, it would be important to assess the depletion efficiency of a CD138 Ab in which the constant region [mouse IgG2a in (17)] has been converted to a different isotype or deleted altogether.

## Concluding Remarks

PCs are key contributors to effective humoral immunity through their robust production of antigen specific Abs. However, it is now readily apparent that these cells do more than just secrete Ig and in fact, play critical roles in normal processes such as hematopoiesis as well as diseases such as EAE. But much remains unknown about these cells in regards to the full spectrum of their regulatory potential. In this respect, the present article has focused on PCs in the adult setting. PCs can be found in the blood of humans as early as 1–5 months of age (74) and are important sources of protective IgA in the gut of newborns (75). Whether or not these cells possess critical Ab-independent functions remains to be determined. Fortunately, both genetic and molecular tools are now available to acutely deplete all PCs and observe the resultant effects on a variety of biological systems. While multiple studies have transcriptionally profiled the PC pool as a whole (17, 76, 77), a further understanding is required in regards to the regulation and extent of functional heterogeneity of PCs both in the naïve state as well as following an infectious or autoimmune response before we will have the capacity to target PCs subsets for a translational outcome.

Using single cell sequencing approaches, it will now be possible to fully visualize the PC landscape and better understand the molecular underpinnings of various subsets of PCs. Analyses of these data may provide important answers to remaining questions such as: What types of B lymphocytes give rise to a particular PC subset? What extracellular signals drive the derivation of a specific type of PC? What core transcriptional elements regulate various PC fractions? Ultimately, this has the potential to lead to the development of PC-targeted immunotherapies for the purposes of modulating specific biological outcomes.

## Author Contributions

PP conceptualized and wrote the manuscript.

### Conflict of Interest

The author declares that the research was conducted in the absence of any commercial or financial relationships that could be construed as a potential conflict of interest.
